# Laboratory medicine perspectives and bio-banking

**DOI:** 10.1186/1753-6561-7-S5-O17

**Published:** 2013-08-30

**Authors:** D Hochstrasser

**Affiliations:** 1 Genetic & Laboratory Medicine Department, Geneva University Hospital, Switzerland

## 

The rapidly evolving fields of genomics, transcriptomics, proteomics and metabolomics can benefit from bio-banks which help to quantify specific molecules, characterize them and to compare profiles. Laboratory medicine utilizes the chemical and physical properties of molecules to analyze them. Mass spectrometry using ionization & fragmentation, magnetic resonance methods and deep sequencing are rapidly evolving in the field of molecular characterization. An introduction was given about working principles of systems such as Triple Quadrupole MS, LTQ Orbitrap, Electron Transfer Dissociation & Collision activated dissociation all of which help in genomic sequencing, peptide sequencing and identification of other probable biomarker molecules.

A properly collected and stored sample will facilitate the future analysis of the genome, transcriptome, proteome and metabolome of the individual. There are issues faced in determining and maintaining optimal conditions for collection and storage of samples. There is also the issue of false positive and false negative results to the tune of 3% in genetic testing. There is a need for issuing frequent advisories regarding technological advances that will help proper collection and storage of samples as it is important in the validation aspect of exposure concept.

